# Sickle cell disease: A distinction of two most frequent genotypes (HbSS and HbSC)

**DOI:** 10.1371/journal.pone.0228399

**Published:** 2020-01-29

**Authors:** Caroline Conceição da Guarda, Sètondji Cocou Modeste Alexandre Yahouédéhou, Rayra Pereira Santiago, Joelma Santana dos Santos Neres, Camila Felix de Lima Fernandes, Milena Magalhães Aleluia, Camylla Vilas Boas Figueiredo, Luciana Magalhães Fiuza, Suellen Pinheiro Carvalho, Rodrigo Mota de Oliveira, Cleverson Alves Fonseca, Uche Samuel Ndidi, Valma Maria Lopes Nascimento, Larissa Carneiro Rocha, Marilda Souza Goncalves

**Affiliations:** 1 Laboratório de Investigação em Genética e Hematologia Translacional, Instituto Gonçalo Moniz, FIOCRUZ-BA, Salvador, Bahia, Brasil; 2 Departamento de Ciências Biológicas, Universidade Estadual de Santa Cruz, UESC, Bahia, Brasil; 3 Laboratório de Pesquisa em Anemias, Departamento de Análises Clínicas e Toxicológicas, Faculdade de Farmácia, Universidade Federal da Bahia, Salvador, Bahia, Brasil; 4 Fundação de Hematologia e Hemoterapia do Estado da Bahia, HEMOBA, Salvador, Bahia, Brasil; Helen Keller International, SIERRA LEONE

## Abstract

Sickle cell disease (SCD) consists of a group of hemoglobinopathies in which individuals present highly variable clinical manifestations. Sickle cell anemia (SCA) is the most severe form, while SC hemoglobinopathy (HbSC) is thought to be milder. Thus, we investigated the clinical manifestations and laboratory parameters by comparing each SCD genotype. We designed a cross-sectional study including 126 SCA individuals and 55 HbSC individuals in steady-state. Hematological, biochemical and inflammatory characterization was performed as well as investigation of previous history of clinical events. SCA patients exhibited most prominent anemia, hemolysis, leukocytosis and inflammation, whereas HbSC patients had increased lipid determinations. The main cause of hospitalization was pain crises on both genotypes. Vaso-occlusive events and pain crises were associated with hematological, inflammatory and anemia biomarkers on both groups. Cluster analysis reveals hematological, inflammatory, hemolytic, endothelial dysfunction and anemia biomarkers in HbSC disease as well as SCA. The results found herein corroborate with previous studies suggesting that SCA and HbSC, although may be similar from the genetic point of view, exhibit different clinical manifestations and laboratory alterations which are useful to monitor the clinical course of each genotype.

## Introduction

Sickle cell disease (SCD) consists of a group of hemoglobinopathies in which individuals inherit hemoglobin variants derived from single point mutations, that causes morphological abnormalities in the red blood cells (RBC) [1]. Sickle cell anemia (SCA) is characterized by the homozygosity for hemoglobin S (HbS) and is the most frequent and severe form of the disease. The point mutation of GAG to GTG in the sixth codon of the β (beta) globin gene (*HBB*), which replaces the glutamic acid for a valine, leading to HbS formation [2]. HbS forms long polymers when the oxygen tension is low, due to the hydrophobic interaction of valine (at 85 position in the globin chain) and phenylalanine (at 88 position in the globin chain) [3]. RBC of SCA individuals are less flexible since the polymers lead to rheological and biochemical changes and hence they impair the blood flow causing vaso-occlusion (VO) [1].

In addition to SCA, hemoglobin SC disease (HbSC) is another genotype of SCD. In this case, individuals inherit HbS in association with hemoglobin C (HbC). The molecular basis of HbSC disease is similar to SCA; however, the point mutation is GAG to AAG, which replaces the glutamic acid for lysine, in the globin chain [2]. The HbC tends to form amorphous aggregate within the RBC which also leads to morphological modifications [4]. In addition, K–Cl cotransporter is also altered in HbSC disease contribute to RBC dehydration, which increases the intracellular hemoglobin concentration, and makes it more dense than HbAA-containing RBC [4].

SCD patients exhibit a wide range of clinical manifestations including acute episodes of pain, pulmonary hypertension (PH), stroke, priapism, leg ulcer, acute chest syndrome (ACS), osteonecrosis and cholelithiasis [1–3]. It is thought that PH, leg ulcer and stroke are associated to the chronic hemolytic feature of SCD, while acute pain crises, osteonecrosis and ACS are associated to VO, which could drive to different subphenotypes [5]. However, this dichotomization is not restricted, very often they overlap and may not be useful for SCA and HbSC individually [5–7]. Moreover, recently it has been suggested that abnormal lipid homeostasis would be surrogate subphenotype, considering the association with both hemolysis and VO [6]. SCA patients usually present clinical events more frequently when compared to HbSC disease, which is considered a milder form of SCD [1,4,8]. Alternatively, retinopathy is more frequently associated to HbSC disease [9].

In addition to different clinical manifestations, laboratory parameters are also important biomarkers useful for the patients’ follow-up due to the possibility to monitor anemia, hemolysis, leukocytosis, endothelial dysfunction and to predict many clinical manifestations [10]. In SCA, RBC count and Hb levels are commonly decreased while complete white blood cells (WBC) counts lactate dehydrogenase (LDH) and reticulocyte counts are increased. Regarding HbSC, RBC counts and Hb levels are usually increased whereas mean corpuscular volume (MCV), mean corpuscular hemoglobin (MCH) and red blood cell distribution width (RDW) are decreased [1,4,8,11]. Laboratory determinations seem to translate the pathophysiological mechanism underlying SCD. Once the HbS alone or in association with HbC forms the polymer, the RBC membrane is also altered [4,5]. Irreversibly sickle RBC are more adherent and can bind to vascular endothelial cells as well as to leukocytes and platelets [12]. This heterogeneous multicellular aggregate leads to physical obstruction of the capillaries driving VO, which is a hallmark of SCD [12]. VO is even heightened due to persistent intravascular hemolysis releasing free heme, hemoglobin (Hb) and arginase which decrease nitric oxide (NO) bioavailability and is directly responsible for endothelial dysfunction [13].

Hemoglobin variants have a high frequency worldwide [14], likewise, SCD is also distributed in several different countries, especially in Africa [15]. Brazilian population bears a heterogeneous genetic background with great admixture, thus SCD prevalence is also diversified through the states, and the incidence of SCD is approximately 1 in 650 newborn babies screened in the state of Bahia [16]. Considering the elevated frequency of hemoglobin variants and prevalence of SCD in our population [17,18], and the peculiarities of SCA and HbSC disease we aimed to investigate the association of classical laboratory parameters and clinical manifestations in each of these SCD genotype.

## Methods

### Study design and casuistic

A cross-sectional study was performed in 181 pediatric SCD children residing in the state of Bahia, Brazil, who were seen at Bahia Hemotherapy and Hematology Foundation (HEMOBA), from October 2016 to September 2017. For inclusion, patients were required to be in steady state, i.e., none had received a blood transfusion 4 months prior to inclusion and no acute events, hospitalization, or infections were reported 3 months prior to blood sampling. Blood samples were taken during a regular clinical visit. Patients with confirmed HbSS or HbSC genotype were included; all the other hemoglobin genotypes were excluded. One hundred and twenty-six patients with SCA (HbSS genotype) aged 14.5 ± 3.5 years of whom 60 (47.6%) were female were enrolled in the study, while 55 with HbSC disease aged 14.1 ± 2.8 years of whom 29 (47.2) were female were also included.

Regarding therapy approaches 62 SCA and 9 HbSC individuals were taking hydroxyurea (HU), moreover, all patients were taking folic acid supplementation. This study received approval from the Institutional Research Board of the São Rafael Hospital (protocol number: 1400535) and was conducted in compliance with the ethical principles established by the revised Declaration of Helsinki. Informed written consent was obtained from each SCD patient’s guardian. When applicable, the children’s acceptance was also registered.

#### Clinical data

Data regarding the occurrence and frequency of previous clinical manifestations were collected using a standardized and confidential questionnaire (self-reported or reported by the parents) at the time of the study enrollment and confirmed by the medical records.

#### Laboratory determinations

Hematological parameters were assessed using a Beckman Coulter LH 780 Hematology Analyzer (Beckman Coulter, Brea, California, USA) and blood smears were stained with Wright’s stain and examined by light optical microscopy. Reticulocytes were counted after staining supravitally with brilliant cresyl blue dye. Hemoglobin patterns were confirmed by high-performance liquid chromatography employing an HPLC/Variant-II hemoglobin testing system (Bio-Rad, Hercules, California, USA).

Biochemical determinations, including lipid profile, total bilirubin and fractions, LDH, iron, hepatic metabolism and renal profile were determined in serum samples using an automated A25 chemistry analyzer (Biosystems S.A, Barcelona, Catalunya, Spain). Ferritin levels were determined using Access 2 Immunochemistry System (Beckman Coulter Inc., Pasadena, California, USA). C-reactive protein (CRP) and alpha-1 antitrypsin (AAT) levels were measured using IMMAGE® Immunochemistry System (Beckman Coulter Inc., Pasadena, California, USA). Determination of NO metabolites (NOm) in serum samples was carried out with the Griess reagent as previously described [19]. Allele-specific PCR was used to investigate the -α^3.7Kb^-thal as previously described [20]. Laboratory parameters were analyzed at the Clinical Analyses Laboratory of the College of Pharmaceutical Sciences (LACTFAR, Universidade Federal da Bahia).

#### Statistical analysis

Statistical analyses were performed using the Statistical Package for the Social Sciences (SPSS) version 20.0 software (IBM, Armonk, New York, USA) and GraphPad Prism version 6.0 (Graphpad Software, San Diego, California, USA), which was also used to assemble the graphs. Baseline values of selected variables are expressed as means with their respective standard variation. We tested each variable distribution employing the Shapiro-Wilk test. The Mann-Whitney *U* test and independent t-test were used to compare the groups according to the normality of the distribution for each variable. Hierarchical clustering of the laboratory parameters was performed using the Ward method and the square Euclidean distance was measured between the variables. All the parameters were standardized by the Z score. P values <0.05 were considered statistically significant.

## Results

### Hematological and biochemical parameters are different in SCA and HbSC disease

In order to first distinguish SCA and HbSC individuals we compared laboratory parameters of both groups. We observed that SCA patients had most prominent anemia, hemolysis and increased leukocyte counts, in addition, α^3.7kb^ thalassemia genotype identification was available for 111 SCA individuals and 49 HbSC patients (Table 1). Moreover, SCA patients also presented increased systemic inflammatory mediators. However, HbSC patients exhibited increased lipid profile as well as renal biomarkers, while NOm levels were decreased (Table 2).

**Table 1:** Hematological characterization of SCA and hemoglobin SC disease patients.

Laboratory parameters	SCA (N = 126)	HbSC (N = 55)	*P* value
	Mean ± SD	Mean ± SD	
Sex, % of females	60 (47.6)	29 (47.2)	-
Age, years	14.5 ± 3.5	14.1 ± 2.8	-
**Hemolysis markers**			
RBC, 10^6^/mL	2.74 ± 0.46	4.26 ± 0.47	**0.000**
Hemoglobin, g/dL	8.47 ± 1.03	11.53 ± 0.89	**0.000**
Hematocrit, %	25.15 ± 3.38	33.09 ± 6.99	0.000*
MCV, fL	92.42 ± 11.63	80.94 ± 5.76	**0.000**
MCH, ρg	31.33 ± 3.97	27.18 ± 2.08	**0.000**
MCHC, g/dL	33.92 ± 1.02	33.56 ± 0.56	0.004*
Reticulocyte count, %	5.16 ± 2.31	3.34 ± 1.28	**0.000**
Reticulocyte counts, /mL	139781 ± 63905	140882 ± 51713	0.636
RDW, %	22.67 ± 3.77	17.19 ± 2.38	**0.000**
Total bilirubin, mg/dL	3.00 ± 1.67	1.31 ± 0.74	**0.000**
Direct bilirubin, mg/dL	0.41 ± 0.16	0.28 ± 0.11	**0.000**
Indirect bilirubin, mg/dL	2.62 ± 1.63	1.09 ± 0.16	**0.000**
LDH, U/L	1250.72 ± 1292.86	599.33 ± 147.34	**0.000**
**Hb pattern**			
HbS, %	83.44 ± 10.29	51.53 ± 4.22	**-**
HbC, %	-	43.37 ± 3.11	**-**
HbF, %	9.05 ± 5.68	1.87 ± 2.20	**0.000**
**Leukocytes**			
WBC /mL	11473 ± 3445	9064 ± 3238	**0.000**
Neutrophils /mL	5585 ± 2638	5083 ± 2585	0.124
Monocytes /mL	1098 ± 582	726 ± 350	**0.000**
Eosinophils /mL	492 ± 488	405 ± 324	0.338
Basophils /mL	93 ± 108	49 ± 75	**0.005**
Lymphocytes /mL	4130 ± 1329	2798 ± 1014	**0.000**
**Platelets**			
Platelet count, x10^3^/mL	422 ± 137	291 ± 102	**0.000**
MPV, fL	7.93 ± 0.86	7.98 ± 1.84	0.840*
PCT, %	0.32 ± 0.10	0.22 ± 0.07	**0.000**
PDW, %	16.29 ± 0.64	17.08 ± 0.81	**0.000**
**α**^**3.7kb**^ **thalassemia genotype**			
Wild-type	86 (77.5%)	42 (85.7%)	-
Heterozygous	17 (15.3%)	4 (8.2%)	-
Homozygous	8 (7.2%)	3 (6.1%)	-

RBC: red blood cells; MCV: mean corpuscular volume; MCH: mean corpuscular hemoglobin; MCHC: mean corpuscular hemoglobin concentration; RDW: red cell distribution width; LDH: lactate dehydrogenase; HbS: hemoglobin S; HbF: fetal hemoglobin; WBC: white blood cell; MPV: mean platelet volume; PCT: plateletcrit; PDW: platelet distribution width. Bold values indicate significance at p<0.05; p-value obtained using Mann-Whitney *U* test.

*p-value obtained using independent t-test.

**Table 2:** Biochemical characterization of SCA and hemoglobin SC disease patients.

Laboratory parameters	SCA (N = 126)	HbSC (N = 55)	*P* value
	Mean ± SD	Mean ± SD	
**Lipid metabolism**			
Total Cholesterol, mg/dL	120.92 ± 24.74	135.00 ± 29.53	**0.002**
HDL-C, mg/dL	35.81 ± 8.72	40.74 ± 11.34	**0.008**
LDL-C, mg/dL	62.10 ± 21.95	72.17 ± 27.64	**0.019**
VLDL-C, mg/dL	22.51 ± 11.26	20.50 ± 6.46	0.984
Triglycerides, mg/dL	109.45 ± 50.48	102.54 ± 32.32	0.905
**Iron metabolism**			
Iron, mcg/dL	111.89 ± 55.03	91.00 ± 32.46	**0.030**
Ferritin, *η*g/mL	259.70 ± 437.89	98.83 ± 100.96	0.287
**Renal profile**			
Urea, mg/dL	17.54 ± 6.54	18.10 ± 5.76	0.130
Creatinine, mg/dL	0.43 ± 0.14	0.62 ± 0.14	**0.000**
Uric Acid, mg/dL	3.81 ± 1.20	4.23 ± 1.08	**0.014**
**Hepatic profile**			
AST, U/L	48.10 ± 18.05	26.69 ± 14.16	**0.000**
ALT, U/L	21.22 ± 14.00	14.89 ± 14.52	**0.000**
GGT, U/L	27.30 ± 22.41	23.19 ± 17.81	0.112
Alkaline phosphatase, U/L	135.53 ± 71.10	180.81 ± 101.85	**0.007**
**Inflammatory profile**			
CRP, mg/L	5.63 ± 6.78	3.87 ± 4.33	**0.001**
AAT, mg/dL	82.49 ± 47.32	69.37 ± 49.32	**0.029**
**NOm, μM**	23.87 ± 14.22	17.50 ± 7.52	**0.000**

HDL-C: high-density lipoprotein cholesterol; LDL-C: low-density lipoprotein cholesterol; VLDL-C: very low-density lipoprotein cholesterol; AST: aspartate amino-transferase; ALT: alanine amino-transferase; GGT: gamma glutamyl-transferase; CRP: C-reactive protein; AAT: alpha-1 antitrypsin. NOm: nitric oxide metabolites. Bold values indicate significance at p<0.05; p-value obtained using Mann-Whitney *U* test. *p-value obtained using independent t-test.

### Severe clinical manifestations are more frequent in SCA

We investigated the frequency of clinical manifestations in each group. SCA patients had most cases of hospital admissions, pneumonia, splenomegaly, stroke, painful crises (PC), vaso-occlusive events (VO), infections, leg ulcer, acute chest syndrome (ACS), bone alterations and cholelithiasis (Table 3). The main cause of hospital admission in both groups was acute pain crises (Table 3); although some patients underwent hospital admission for more than one cause. Comparing SCA and HbSC disease, we found statistical significance for PC, VO and cholelithiasis. Considering the physiopathological relevance of both PC and VO for the pathogenesis of SCD we decided to further investigate which laboratory parameters would be associated by comparing the groups who had the clinical manifestation with those who had not.

**Table 3:** Frequency of clinical events in SCA and hemoglobin SC disease patients.

Clinical manifestation	SCA (N = 126)	HbSC (N = 55)	*P* value
Hospital admissions	118	40	**-**
Causes of hospital admissions*			
Acute pain crises	93	29	
Pneumonia/ACS	36	10	
Infections	32	13	
Surgery	5	-	
Neurology	4	1	
Cardiology	1	-	
Angiology	1	-	
Nephrology	1	-	
Other clinical manifestation	17	12	
Infections	86	31	0.128
Painful crises	78	46	**0.005**
Pneumonia	69	24	0.195
Splenomegaly	59	26	1.00
Vaso-occlusive events	46	9	**0.008**
Cholelithiasis	39	7	**0.014**
Acute chest syndrome	33	8	0.086
Stroke	13	2	0.155
Leg ulcer	12	7	0.600
Bone alterations	10	4	1.000

Bold values indicate significance at p<0.05. P-value obtained with Fisher’s exact test.

*Of note: some patients underwent hospital admission due to multiple clinical complications.

### Hematological parameters are associated with clinical manifestations in HbSC disease

**Fig 1:**
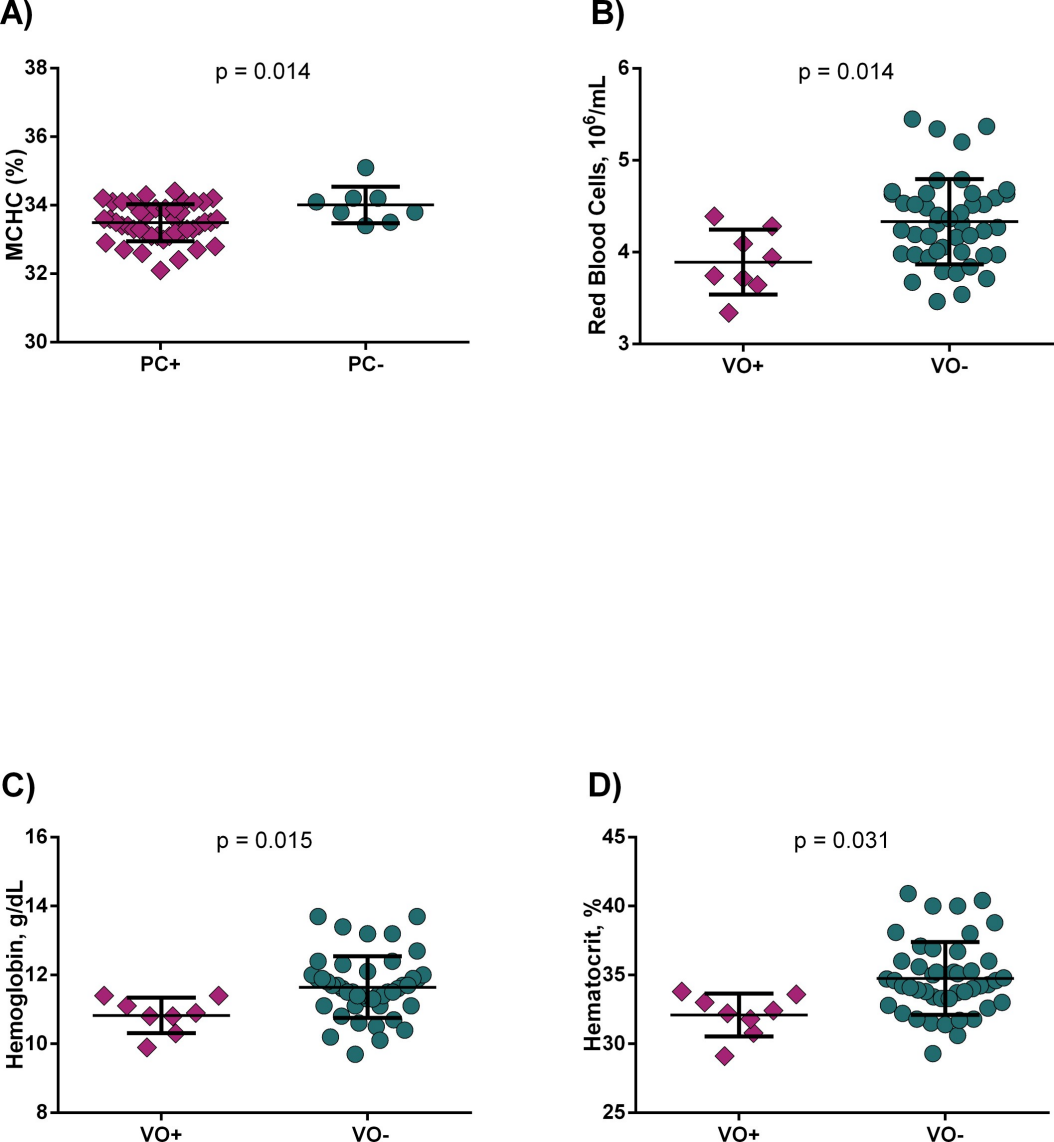
Hematological laboratory parameters are associated to clinical manifestations in HbSC disease. A) Patients with previous history of painful crises (PC) had decreased MCHC; B) Patients with previous history of vaso-occlusion (VO) had decreased red blood cell counts; C) hemoglobin and D) hematocrit levels. p-value obtained using Mann-Whitney *U* test.

Although HbSC individuals presented less complicated anemia and hemolysis, hematological parameters were associated to clinical manifestations. Patients with HbSC and previous history of PC had decreased mean corpuscular hemoglobin concentration (MCHC) (Fig 1A). HbSC patients with previous history of VO exhibited decreased RBC counts (Fig 1B), as well as Hb (Fig 1C) and Ht (Fig 1D) levels.

Considering the association between hematological parameters and clinical manifestations in HbSC disease we also performed a multivariate linear regression model with pain crises as dependent variable. Our model has shown that MCHC, Hb and Ht were independently associated with pain crises in HbSC disease (Table 4).

**Table 4:** Multivariate linear regression model of history of pain crises in association with confounding variables in hemoglobin SC disease and SCA patients.

Independent variables	Dependent variable	β	p-value	R^2^	p-value of the model
**HbSC**					
RBC, 10^6^/mL		-0.201	0.343		
MCHC, %	Pain crises	-1.274	**0.003**	**0.223**	**.015**
Hemoglobin, g/dL		4.284	**0.024**		
Hematocrit, %		-4.066	**0.029**		
**SCA**					
RBC, 10^6^/mL		0.064	0.507		
Reticulocytes, /mL		0.171	0.073		
CRP, mg/L	Pain crises	0.106	0.249	**0.125**	**.012**
AAT, mg/dL		0.120	0.194		
NOm, μM		-0.190	**0.046**		

R^2^: coefficient of determination; β: coefficient of regression.

### Hematological and inflammatory determinations are associated to clinical manifestations in SCA

**Fig 2:**
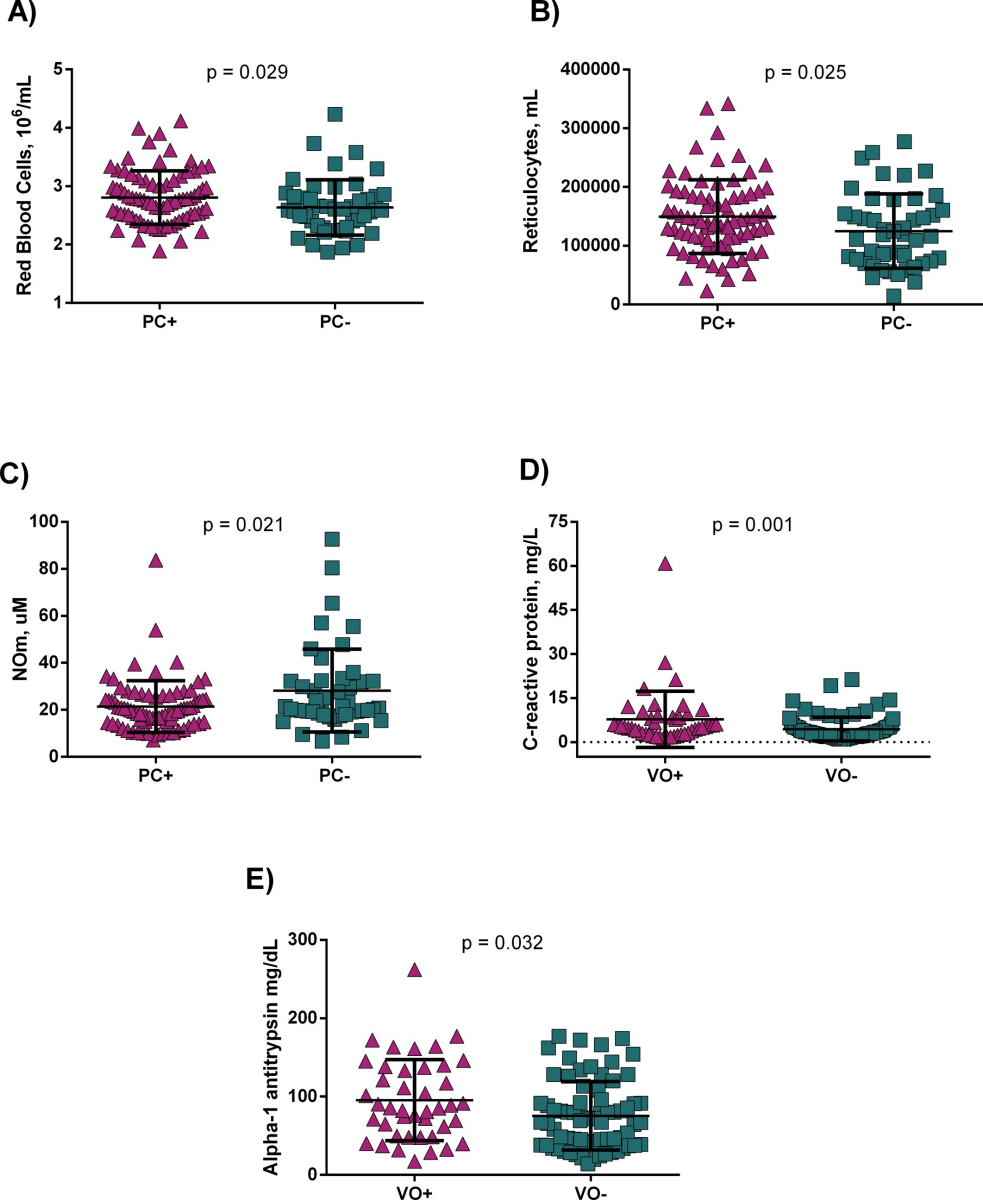
Hematological and inflammatory laboratory parameters are associated to clinical manifestations in SCA. A) Patients with previous history of painful crises (PC) had increased red blood cells and B) increased reticulocyte counts, and C) decreased nitric oxide metabolites (NOm). D) Patients with previous history of vaso-occlusion (VO) had increased C-reactive protein and E) Alpha-1 antitrypsin levels. p-value obtained using Mann-Whitney *U* test.

SCA patients exhibit the most severe form of SCD. PC was associated to increased RBC (Fig 2A) and reticulocyte (Fig 2B) counts; in addition to decreased NOm levels (Fig 2C). VO also seems to be associated to a chronic inflammatory response since patients with previous history of VO had increased C-RP (Fig 2D) and AAT (Fig 2E) levels.

Considering the association between hematological and inflammatory parameters and clinical manifestations in SCA we also performed a multivariate linear regression model with pain crises as dependent variable. Our model has shown that NOm was independently associated with pain crises in SCA (Table 4).

### Cluster analysis reveals different groups of laboratory parameters in SCA and HbSC disease

We also tested which laboratory parameters would be clustered in each genotype. In HbSC disease cluster analysis reveals that in the distance 25 two large groups were formed. In the upper part of the cluster, in the distance 17 two other groups were formed. The upper, in the distance 7, included PDW, MPV, NOm, triglycerides and VLDL-C while the lower, in the distance 15, included AST, ALT, GGT, ferritin, LDH, MCV, MCH, total bilirubin, indirect bilirubin, MCHC and HbF. In the distance 20 two other groups were formed, the first, in the distance 16, included Hb, Ht, RBC, iron, uric acid, creatinine, AAT, total cholesterol, LDL-C, HDL-C, direct bilirubin, basophils and urea; while in the distance 17 a group was formed consisted of reticulocytes, RDW, CRP, eosinophils, alkaline phosphatase, leukocytes, neutrophils, monocytes, platelets, PCT and lymphocytes (S1 Fig).

Regarding SCA, cluster analysis reveals that in the distance 25 two large groups were formed. In the distance 19 two other groups were formed, the upper in the distance 13 included total bilirubin, indirect bilirubin, AST, ALT, MCHC, RDW, lymphocytes, direct bilirubin, VLDL-C, triglycerides, NOm and LDH. The other group in the distance 14 included platelets, PCT, reticulocytes, leukocytes, neutrophils, monocytes, eosinophils, basophils, MPV, total cholesterol, LDL-C, urea, CRP, PDW, alkaline phosphatase, GGT, AAT, HDL-C and ferritin. The lowest cluster in the distance 14 was consisted of MCV, MCH, iron, creatinine, uric acid, Hb, Ht, RBC and HbF (S2 Fig).

## Discussion

Although the molecular basis of each SCD genotype is clear, the mechanisms contributing to clinical manifestations and to the maintenance of inflammation are not fully understood. This study was conducted to perform a wide characterization of SCD assessing the two most frequent genotypes.

Baseline laboratory characteristics of SCA patients are consistent with previous evaluation, revealing anemia, hemolysis, leukocytosis and the increase of systemic inflammation [8,21,22]. Importantly, total leukocyte counts above 15,000 cells/mL^3^ as well as low HbF levels were associated with an increased risk of early death [21]. Likewise, intravascular hemolysis is also associated to the severity of clinical outcomes [23]. Acute phase proteins, such C-RP and AAT, are produced especially by the liver during infections or inflammatory conditions [24]. C-RP and AAT levels were shown to be elevated among SCD patients even during steady-state [25]. Altogether, our data reinforce the notion that SCA is the most severe SCD genotype. Laboratory investigation of HbSC individuals revealed increased lipid, creatinine and uric acid levels as well as decreased NOm. Our findings are in agreement with previous laboratory profile of HbSC disease [26], including increased creatinine levels [27] and increased total cholesterol, HDL-C and LDL-C as well as decreased NOm determinations [8]. This lipid profile among HbSC individuals has also been show in other populations [28].

Clinical events in SCD are driven by the pathophysiological mechanism of VO. Indeed, all the clinical manifestations investigated were more prevalent in the SCA group than in HbSC disease. This is in agreement with previous clinical and laboratory characterization of SCA and HbSC disease patients [8], which corroborate that SCA is more severe. An evaluation of a cohort of ten years has also found that the onset of the complications was earlier in SCA compared to HbSC patients, especially for painful crises and acute chest syndrome [29]. Acute pain crises are the most common cause of hospitalization among SCD patients. In our population we have found that the most frequent cause of hospital admission was acute pain crises in SCA and HbSC disease, which was also observed in different populations where the major cause of hospital admission was acute painful episodes accounting for 94.6% of the admissions [30]. A survey carried out in England has identified that primary diagnoses for admission was sickle cell crises, followed by acute lower respiratory tract infection and asthma [31]. In addition, cholelithiasis is a frequent complication in SCD patients due to the ongoing hemolysis which results in the production of large amounts of bilirubin, which is conjugated in the liver and its accumulation, may form calcium bilirubinate gallstones [32]. Collectively, these findings suggest that regardless of the SCD genotype, pain crises are the most important clinical event patients have experienced.

PC and VO were statistically different when SCA was compared to HbSC disease, which lead us to examine laboratory parameters in each group. Hematological and inflammatory parameters were shown to be associated with PC and VO in both HbSC disease and SCA.

Reticulocyte and RBC counts as well as MCHC levels were associated with pain crises in our SCA and HbSC patients which allow us to suggest that hemolysis and anemia are thought to contribute to clinical outcome in SCD. Reticulocytosis has been associated to increase in hospitalization during the first three years of life of children with SCA [33]. Moreover, an extensive hemolysis evaluation has shown that absolute reticulocyte counts and reticulocyte percentage had a strong inverse correlation with mean RBC survival [34]. Correspondingly, HbF levels were shown to be decreased in children with SCA with absolute reticulocyte counts greater than 200 000 cells/mL [35]. Altogether, these findings suggest that hemolysis may be measured through routine hematological evaluation, such as reticulocyte counts, which is important to monitor the patient outcome.

Several pain mediators have been described in SCD such as interleukin-1, bradykinin, histamine, substance P and calcitonin gene related peptide [36]. Pain crises in SCD may be acute, chronic or a combination of both and is usually secondary to vaso-occlusion [36]. Hemolysis leads to endothelial dysfunction since it causes the release of Hb and heme which limits NO bioavailability as well as arginase, which consumes L-arginine, decreasing NO levels even more and contributing to VO [13]. Thus, the association of both pathophysiological mechanisms to the triad of factors (VO, inflammation and nociception) may help to initiate the acute painful crises [37]. Abnormal lipid homeostasis has also been associated with decreased NOm levels [6].

Chronic inflammatory response is a hallmark of SCD influenced by leukocytes, platelets [38], intravascular hemolysis and innate immune response [13] and increased pro-inflammatory mediators [39]. Our cohort of patients with previous history of VO exhibited laboratory parameters associated to anemia and systemic inflammation. Increased AAT levels were found to be associated to infections, gallstones and blood therapy in SCD [40]; moreover, C-RP levels were progressively increasing as SCA severity score was higher [41]. Our findings are in agreement with the pathophysiological mechanism of VO due to i) heightened ability of sickle RBC to adhere to the vascular endothelium and promote activation of endothelial cells and leukocytes and ii) sickle RBC have the lifespan shortened which also contributes to anemia [42].

Cluster grouping is a very useful approach to identify biomarkers of SCD severity [43]. We designed a cluster analysis in order to group the laboratory parameters of each genotype. Cluster analysis among HbSC disease patients has shown 4 different cluster agglomerations with participation of hemolytic and endothelial dysfunction parameters in the two first, as well as hematological and inflammatory parameters in the latter two. Contrarily, cluster analysis among SCA patients has shown 3 different cluster agglomerations with participation of hemolytic parameters in the first cluster, leukocytes, lipid metabolism and inflammatory parameters in the second cluster and markers of iron metabolism and anemia in the last cluster.

HbSC patients are known to exhibit a phenotype with increased viscosity [4,9,27] which may be corroborated by our findings of clustering hemolysis and endothelial dysfunction markers in the similar groups in these genotype [8,10,13]. As they also present less severe anemia, clustering of hematological and inflammatory markers in similar groups is in agreement with the literature [8,10,13]. SCA patients present the most severe phenotype of SCD and our cluster analysis demonstrate tree groups: hemolysis, inflammation and anemia. These markers are suggestive of the main underlying pathophysiological mechanisms of the disease, which often overlap [1]. In the first cluster the association of NOm and hemolytic markers reinforces the role of endothelial dysfunction [13,44], while in the second the association of leukocytes counts and CRP and AAT highlights the role of inflammation [40,41] and in the last cluster, grouping of RBC counts along with Hb, Ht and iron levels suggest the importance of anemia [21,22]. Curiously HbF was differentially clustered in each genotype. In HbSC patients HbF was clustered along with biomarkers of hemolysis (LDH, AST, indirect bilirubin), while in SCA it was clustered along with biomarkers of anemia (RBC counts, Hb, Ht, MCV and MCH levels). HbF levels are one of the most important biomarker for disease prognostic in SCD [10,35,45], altogether our results suggests that different mechanisms may be associated with HbF in the different SCD genotypes. The different classification of the same laboratory parameters on HbSC disease and SCA suggests that, indeed, the same measurement obtained with one genotype may have a different relevance when compared with the other genotype of the same disease.

Our data suggest that SCA patients exhibit increased hemolysis and inflammatory parameters as well as more clinical complications. In addition, HbSC patients exhibit altered lipid metabolism and milder hemolysis. Moreover, laboratory parameters are also important to monitor the disease. Of note, it is important to point that our cohort is composed by pediatric patients and the clinical course is usually more complicated in the greater ages. Nevertheless, our findings support the differences between SCA and HbSC disease that should be taken into account when considered clinical management.

## Supporting information

S1 Fig Cluster analysis of laboratory biomarkers among HbSC disease. Dendogram demonstrating cluster agglomeration of laboratory parameters in the group of patients with HbSC disease. The interval was measured by the square Euclidean distance and measurements were standardized by the Z score.
(TIF)

S2 Fig Cluster analysis of laboratory biomarkers among SCA patients. Dendogram demonstrating cluster agglomeration of laboratory parameters in the group of patients with SCA. The interval was measured by the square Euclidean distance and measurements were standardized by the Z score.
(TIF)
