# Supplementary figures and images for: Sickle cell disease: A distinction of two most frequent genotypes (HbSS and HbSC)
Source: PLoS One. 2020 Jan 29;15(1):e0228399. doi: 10.1371/journal.pone.0228399 (PMC6988974; doi:10.1371/journal.pone.0228399)

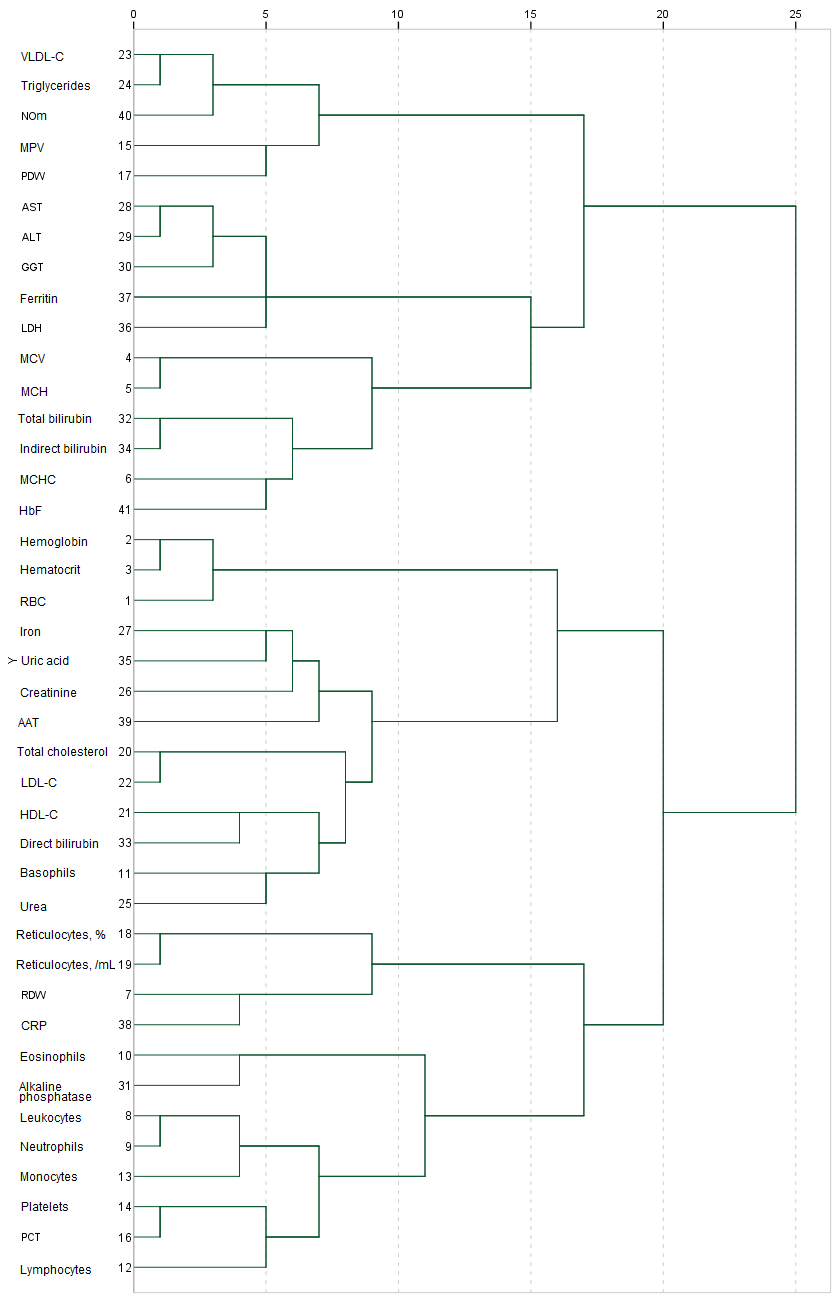

Supplement: S1 Fig — Dendogram demonstrating cluster agglomeration of laboratory parameters in the group of patients with HbSC disease. The interval was measured by the square Euclidean distance and measurements were standardized by the Z score. (TIF) [file pone.0228399.s001.tif]

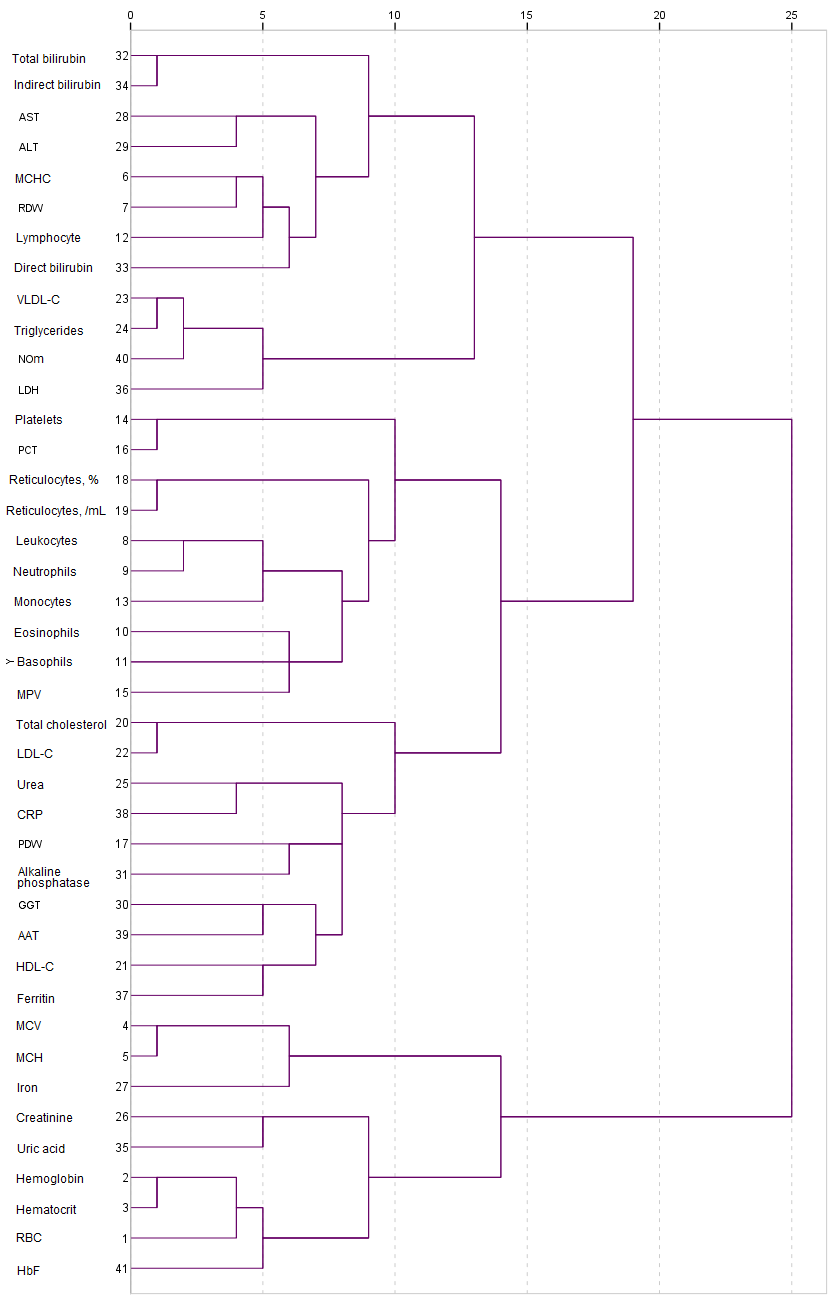

Supplement: S2 Fig — Dendogram demonstrating cluster agglomeration of laboratory parameters in the group of patients with SCA. The interval was measured by the square Euclidean distance and measurements were standardized by the Z score. (TIF) [file pone.0228399.s002.tif]
